# Aerobic Exercise Training in Patients With mtDNA-Related Mitochondrial Myopathy

**DOI:** 10.3389/fphys.2020.00349

**Published:** 2020-05-21

**Authors:** Tina Dysgaard Jeppesen

**Affiliations:** Copenhagen Neuromuscular Clinic, Department of Neurology, Rigshospitalet, University of Copenhagen, Copenhagen, Denmark

**Keywords:** mitochondrial DNA, mitochondrial myopathies, treatment, training, oxidative capacity

## Abstract

In patients with mitochondrial DNA (mtDNA) mutation, a pathogenic mtDNA mutation is heteroplasmically distributed among tissues. The ratio between wild-type and mutated mtDNA copies determines the mtDNA mutation load of the tissue, which correlates inversively with oxidative capacity of the tissue. In patients with mtDNA mutation, the mutation load is often very high in skeletal muscle compared to other tissues. Additionally, skeletal muscle can increase its oxygen demand up to 100-fold from rest to exercise, which is unmatched by any other tissue. Thus, exercise intolerance is the most common symptom in patients with mtDNA mutation. The impaired oxidative capacity in skeletal muscle in patients with mtDNA mutation results in limitation in physical capacity that interferes with daily activities and impairs quality of life. Additionally, patients with mitochondrial disease due to mtDNA mutation often live a sedentary lifestyle, which further impair oxidative capacity and exercise tolerance. Since aerobic exercise training increase mitochondrial function and volume density in healthy individuals, studies have investigated if aerobic training could be used to counteract the progressive exercise intolerance in patients with mtDNA mutation. Overall studies investigating the effect of aerobic training in patients with mtDNA mutation have shown that aerobic training is an efficient way to improve oxidative capacity in this condition, and aerobic training seems to be safe even for patients with high mtDNA mutation in skeletal muscle.

## Introduction

**FIGURE 1:**
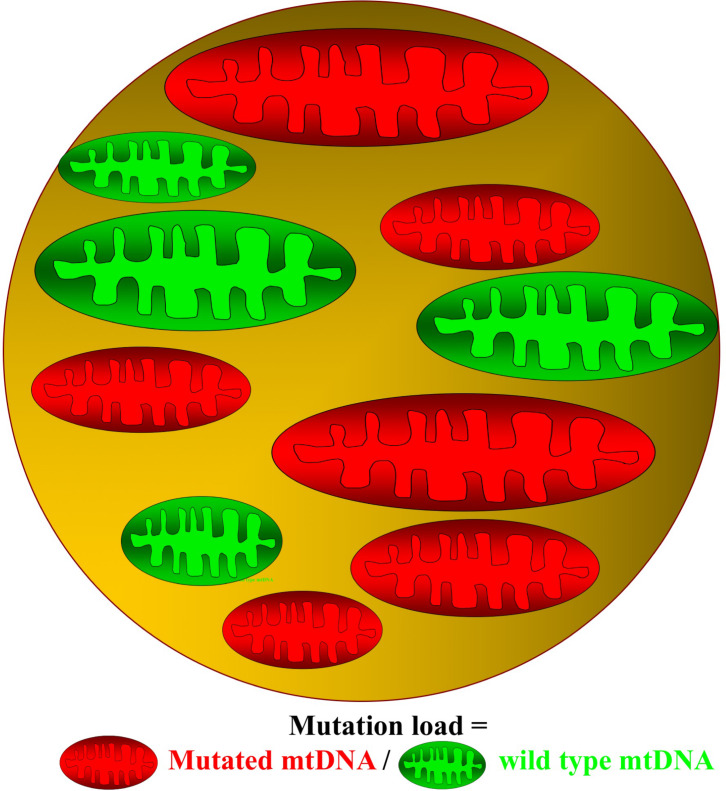
Cell with mutated and wild-type mitochondrial DNA. In patients with mitochondrial DNA (mtDNA), mutations contain both mutated (red) and wild-type (green) mitochondria. The ratio between mutated mtDNA and wild-type mtDNA denotes the mutation load of the cell.

**FIGURE 2:**
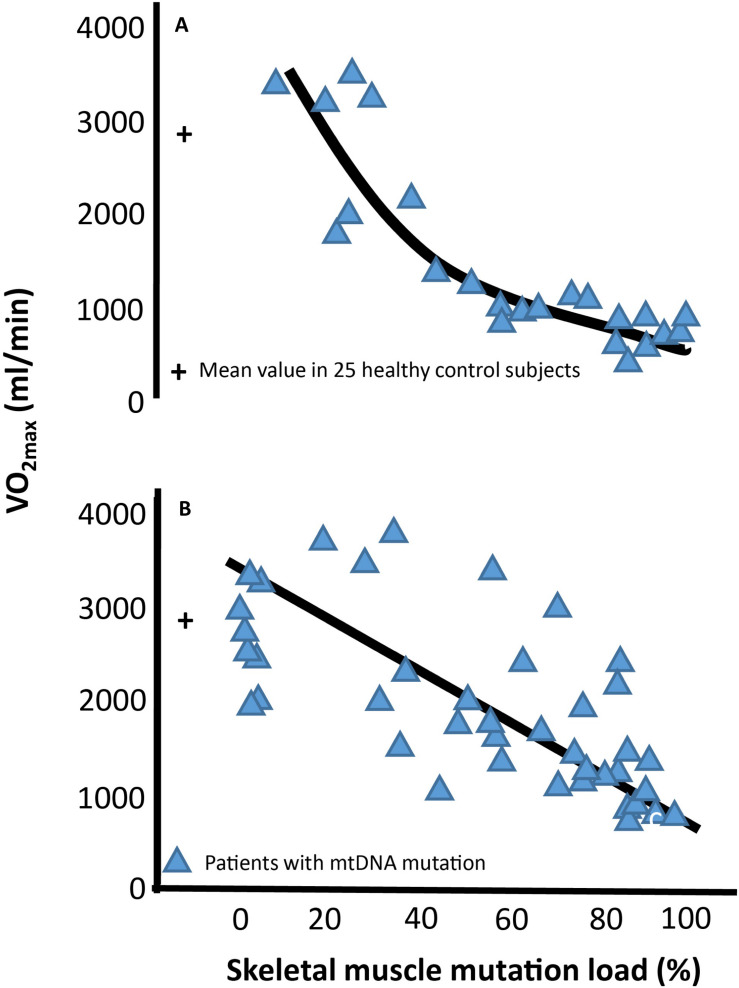
Association between maximal oxidative capacity and skeletal muscle mitochondrial DNA mutation load Correlation between level of percentage mitochondrial DNA (mtDNA) mutation load in skeletal muscle and maximal oxygen uptake (VO_2max_) in 24 subjects with different mtDNA mutation **(A)** and 51 subjects with the 3,243 A > G point mutation of mtDNA **(B)**. Blue triangles each represent a subjects with mtDNA mutation. The cross at 0% muscle mutation represents the mean ±SE of 25 age- and gender matched healthy subjects.

Mitochondria are small dynamic organelles that, with the exception of mature red blood cells, exist in all cells throughout the human body. Mitochondria contain the electron transport chain where ATP is produced through conversion of oxygen into water. Besides energy production, mitochondria are essential for cell signaling particularly apoptosis, and mitochondria host several metabolic pathways. Given these fundamental roles, it is not hard to imagine that defects in mitochondrial function can have catastrophic consequences for the cell. In patients with mitochondrial DNA (mtDNA) mutations, only a fraction of the mtDNA harbors mutation (Figure 1). Studies have demonstrated that oxidative capacity and the mtDNA mutation load of the tissue correlates, at least when measured in skeletal muscle (Figure 2; Tatuch et al., 1992; Jeppesen et al., 2003, 2006a; Frederiksen et al., 2006), indicating a close relationship between mtDNA mutation and the impact of mitochondrial dysfunction on different tissues.

**FIGURE 3:**
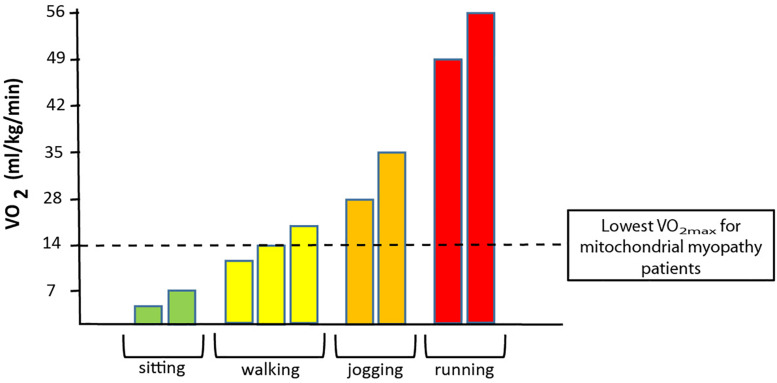
Association between oxygen uptake and different physical activities. The graph demonstrates the range of oxygen demand demonstrated in VO_2_ (oxygen uptake) during no activity (sitting) to walking (slow to brisk walking speed), jogging and to running at maximal speed. The dotted black line denotes the maximal oxidative capacity that is found in patients with a high level of mtDNA mutation load in skeletal muscle.

In patients with mtDNA mutations, mutation load is often high in skeletal muscle, and since oxidative demand is higher in skeletal muscle than in any other tissue, exercise intolerance is the most common symptom in patients with mtDNA mutations. This exercise intolerance relates to a low oxidative capacity in many patients, where the maximal oxidative capacity is lower than what is needed for cycling and jogging (Figure 3). Thus, the low oxidative capacity seriously interferes with trivial daily activities, which reduces quality of life and results in a sedentary lifestyle that increases the risk of secondary diseases such as diabetes and cardiovascular diseases. Muscle is a highly adaptable tissue that responds to changes in nutrition, hormones, and training (Booth and Watson, 1985; Babij and Booth, 1988). Aerobic training induces mitochondrial function and volume density (Holloszy, 1975; Andersen and Saltin, 1985; Hoppeler and Fluck, 2003), and result in higher anaerobic threshold and enhanced functional work capacity in both healthy individuals (Holloszy, 1967, 1975; Andersen and Saltin, 1985; Turner et al., 1997; Hoppeler and Fluck, 2003; Tarnopolsky et al., 2007) and patients with chronic disorders (Bruce and Hawley, 2004; Sveen et al., 2007, 2008; Cornelissen et al., 2013; Di Meo et al., 2017). Taken that quality of life is reduced due to impaired exercise tolerance (Figure 3) and the increased risk of secondary diseases that a sedentary lifestyle results in, studies have focused on using aerobic training as treatment for impaired oxidative capacity in patients with mtDNA mutations. In the following, the physiological consequences of mtDNA mutations and effect of aerobic training is described.

## The Impact of Aerobic Training on Oxygen Delivery and Uptake

**FIGURE 4:**
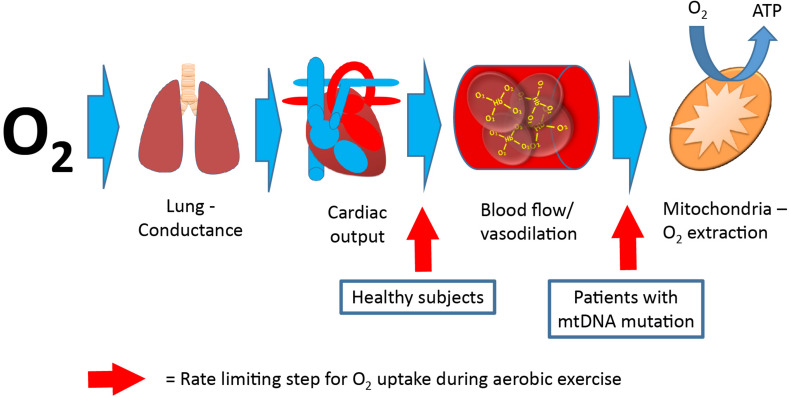
Pathway for oxygen, from air to skeletal muscle. Oxygen uptake from lungs to final destination, the mitochondria. The rate-limiting step (red arrow) for maximal oxygen uptake is oxygen delivery (cardiac output) in healthy subjects, whereas the rate-limiting step for maximal oxygen uptake is oxygen extraction in patients with mitochondrial myopathy (final step).

**FIGURE 5:**
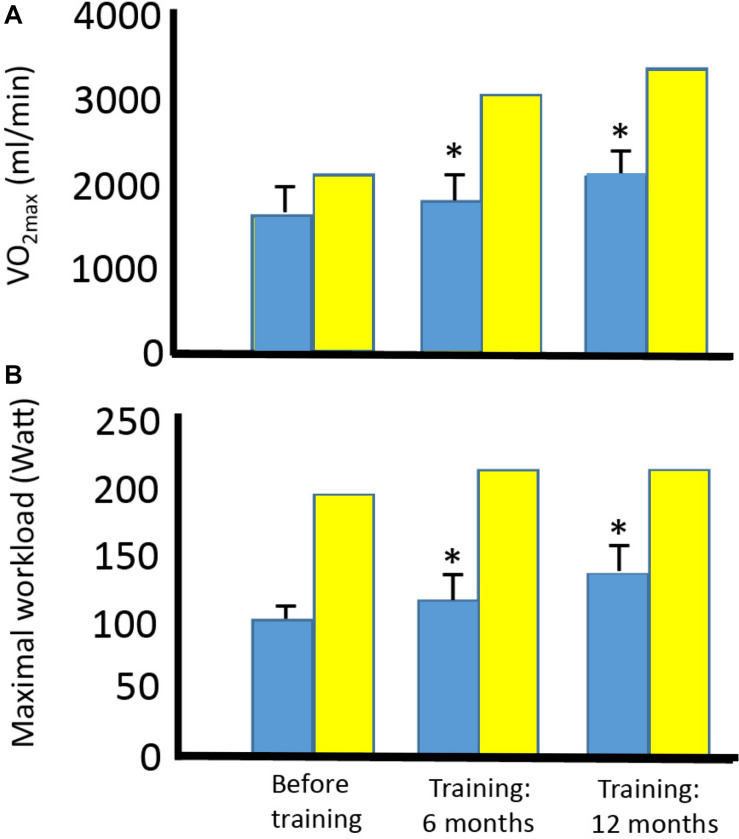
Effect of aerobic training on maximal oxygen uptake and maximal workload in four patients with mtDNA mutation and one healthy subject. Maximal oxygen uptake **(A)** and maximal workload **(B)** before training and after 6 and 12 months of training in four patients with different mtDNA mutations (blue bars) and one healthy subject (yellow bar). *Different compared to before training (*p* < 0.05). ^#^Different compared to before training and after 6 months of training.

Maximal oxygen uptake (VO_2__max_) is used as a parameter for exercise capacity, since oxygen uptake from rest to maximal exercise is attributed to skeletal muscle oxygen uptake alone (Mitchell et al., 1958; Hoppeler and Weibel, 1998; di Prampero, 2003). VO_2__max_ depends on the ability to deliver oxygen from air to blood (lung conductance), the capacity to deliver oxygen (cardiac output), and the capacity to extract oxygen and produce ATP (Saltin, 1986; Bangsbo et al., 2000). In healthy individuals, cardiac output is the rate-limiting step for VO_2__max_ (Saltin, 1986; Berglund and Ekblom, 1991; Saltin and Strange, 1992; Figure 4). In contrast, mitochondrial capacity and function seems to be the rate-limiting step for VO_2__max_ in patients with mtDNA mutations (Haller et al., 1978; Haller and Lewis, 1984; Figure 4). This notion is emphasized by a consistent finding of inverse correlation between VO_2__max_ and mtDNA mutation load in skeletal muscle of patients with mtDNA mutation (Jeppesen et al., 2003, 2006a; Taivassalo et al., 2003; Figure 2). This finding indicates that VO_2__max_ is a good marker of the oxidative dysfunction caused by mtDNA mutation. Since endurance training increases mitochondrial function in healthy individuals, studies have investigated if aerobic training could be used as treatment for the impaired oxidative capacity and exercise intolerance in patients with mtDNA mutation (Taivassalo et al., 1996, 1998, 1999, 2001, 2006; Siciliano et al., 2000, 2012; Cejudo et al., 2005; Jeppesen et al., 2006b, 2009a). Studies have investigated 8–48 weeks of aerobic training (treadmill and cycle training) in 4–20 patients with different mtDNA mutations, exercising 30–45 min three to five times per week at workloads of 60–85% of VO_2__max_. The conclusion from the different studies was overall the same: Patients with mtDNA mutations, irrespective of mutation type are able to increase VO_2__max_ to the same extent as that found in healthy subjects (24%; 20–28%) (Taivassalo et al., 1998, 2001, 2006; Siciliano et al., 2000, 2012; Cejudo et al., 2005; Jeppesen et al., 2006b, 2009a; Adhihetty et al., 2007; Porcelli et al., 2016; Figure 5).

**FIGURE 6:**
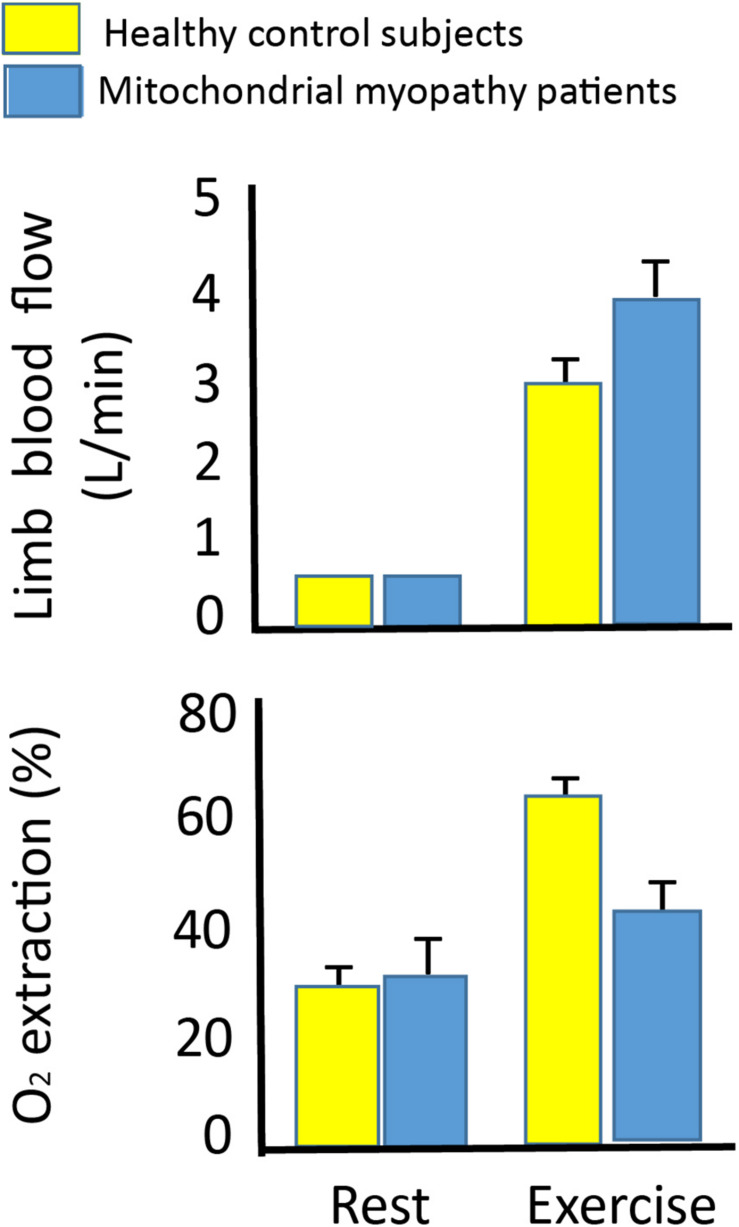
Leg blood flow and oxygen extraction at rest and during leg exercise in 10 patients with mtDNA mutation and 10 healthy subjects. Leg blood flow **(upper graph)** and oxygen extraction **(lower graph)** in 10 patients with mtDNA mutation (blue bars) and 10 healthy subjects (yellow bars) at rest and during one-legged knee exercise at maximal workload (27 ± 3 vs. 55 ±6 W). There was a higher leg blood flow in the patients with mtDNA mutation compared to healthy subjects, while oxygen extraction did not change from rest to exercise indicating a hyperemic response to exercise in patients with mtDNA mutation. *Different from healthy subjects (*p* < 0.05).

Oxygen delivery during exercise is strictly regulated. The relationship between oxygen delivery (cardiac output) and utilization in healthy individuals is 5 L of blood for every 1 L of VO_2_ irrespective of age, gender, or physical condition (Andersen and Saltin, 1985; Saltin and Strange, 1992). Cardiac output is regulated by different factors, including muscle (local vasomotor effect), central factors like systemic vasoconstriction and respiratory pump, systemic vasodilatation, and CNS stimuli of heart rate and contractility and redistribution of blood from non-active muscle cells (Andersen and Saltin, 1985; Saltin and Strange, 1992; Bangsbo et al., 2000). Since the rate-limiting factors for VO_2__max_ in healthy individuals is cardiac output (Figure 4), exercise-induced increase in VO_2__max_ is related to improvement of cardiac output and, to a much lesser extent, increased oxygen extraction for exercising muscle (Saltin, 1986; Bangsbo et al., 2000). Larsson et al. (1964) were the first to demonstrate arterialization of venous blood from contracting muscle during cycle exercise in patients with mtDNA mutation. Since then, many studies have confirmed that delivery of oxygen does not seem to be rate-limiting for VO_2__max_, and instead, there is a hyperemic response to exercise in patients with mtDNA mutations (Bank and Chance, 1994; Ozawa et al., 1995; Abe et al., 1997; Bank and Chance, 1997; Taivassalo et al., 2002). The only study that has investigated oxygen delivery and consumption directly in these conditions showed that while oxygen extraction was normal at rest, patients with mtDNA mutation were unable to increase extraction levels during exercise, along with findings of a workload-adjusted maximal exercise leg hyperemia up to ∼twofold (mean 65%) higher than that found in healthy controls (Jeppesen et al., 2012; Figure 6). Interestingly, the hyperemic response seemed to be induced by an excessive unloading of vasodilating substance ATP (Jeppesen et al., 2012). Four studies have investigated the physiological mechanisms behind increases in VO_2__max_ with aerobic exercise in patients with mtDNA mutation, and interestingly, the driving factors for improvement in VO_2__max_ was different than that found in healthy subjects (Taivassalo et al., 1998; Taivassalo et al., 2001, 2006; Porcelli et al., 2016). The studies demonstrated that cardiac output did not increase linearly to the increase in VO_2__max_ as seen in healthy subjects. Instead, cardiac output either stayed unchanged or only improved, to a small extent, despite a substantial increase in VO_2__max_. With near-infrared spectroscopy (Porcelli et al., 2016), ^31^P-magnetic resonance spectroscopy (^31^P-MRS) (Taivassalo et al., 1998, 2001), and calculation a-vO_2_ extraction (Taivassalo et al., 1998, 2001, 2006; Porcelli et al., 2016), authors demonstrated that the improvement in VO_2__max_ in patients with mtDNA mutation was driven by an increase in mitochondrial capacity related to training-induced improvement in half-time recovery of ADP, the initial rate of phosphocreatine resynthesis, and maximum rate of ATP synthesis (Taivassalo et al., 1998, 2001). In line with these findings, another study demonstrated, with near infrared-spectroscopy technique, that the mismatch between oxygen delivery and oxygen consumption was partly ameliorated after aerobic exercise training in patients with mtDNA mutations (Porcelli et al., 2016). These findings indicate that improvement in oxygen extraction from exercising skeletal muscle may dominate the training response in patients with mtDNA mutations. Additionally, Porcelli et al. (2016) demonstrated that in patients with mtDNA mutations that had impaired pulmonary VO_2_ pre-training, pulmonary VO_2_ kinetics increased after 12 weeks of aerobic training. Since VO_2_ kinetics reflect performance of skeletal muscle oxidative metabolism, the improvements in pulmonary VO_2_ kinetics indicate that some of the patients with mtDNA mutation obtained lower O_2_ deficit and higher exercise tolerance after 12 weeks of moderate intensity cycle training (Porcelli et al., 2016).

**FIGURE 7:**
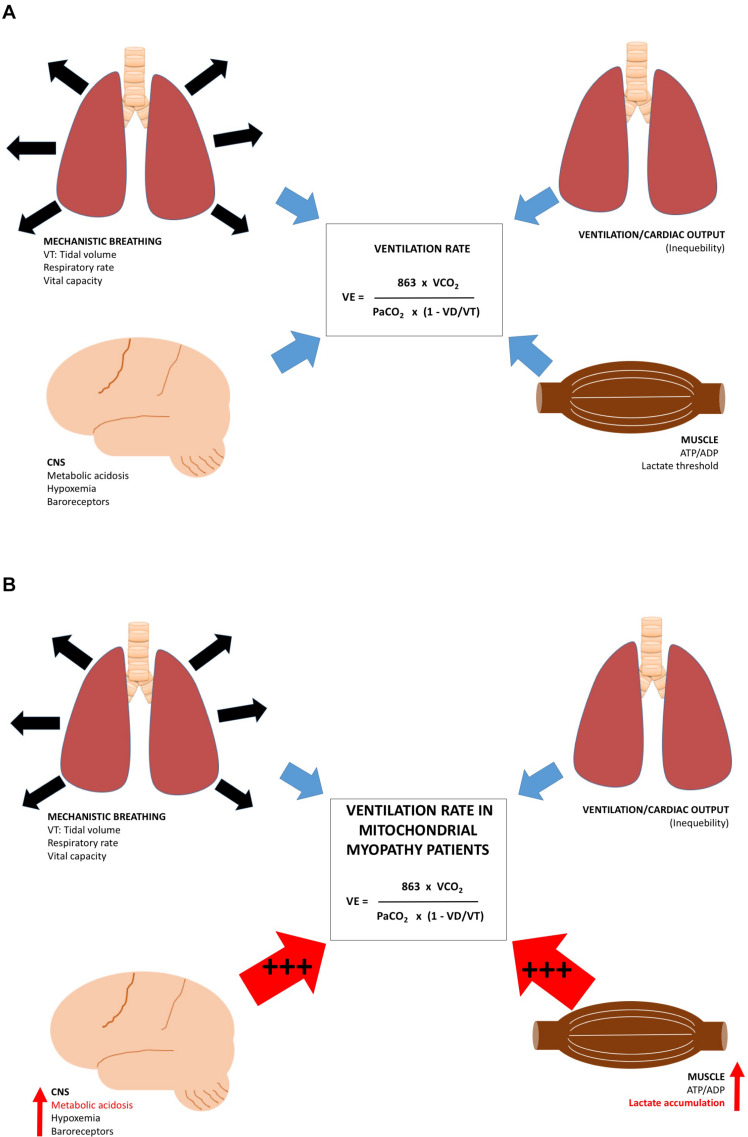
Regulating factors for ventilation rate at rest and during exercise. Different regulating factors for rate of ventilation. (1) factors responsible for lung function, (2) Central nervous system, (3) relationship between cardiac output, and oxygen uptake in the lungs, and (4) ATP/ADP, lactate and pH in skeletal muscle in **(A)** healthy subjects and **(B)** patients with mtDNA mutation. During exercise, patients with mtDNA mutation have an excessive metabolic acidosis increasing the CNS-mediated drive on ventilation rate (+++), the ATP/ADP ratio (+++), pH drop, and lactate concentration accumulate (+++), which result in increased skeletal muscle drive on ventilation rate.

The rate of pulmonary ventilation (VE) increases linearly with exercise intensity until the VE threshold is reached (Goldstein et al., 1975; Martin and Weil, 1979; Davis et al., 1982). From this point, VE exceeds oxygen uptake, and lactate is accumulated (Davis et al., 1983). VE is tightly regulated by areas in the central nervous system, peripheral nervous system, including feedback from mechanical breathing pattern, arterial carbon dioxide, and oxygen tension (Dempsey et al., 1985; Forster and Pan, 1988), and a direct feedback from muscle due to a decrease in ATP:ADP ratio, lactate accumulation, and fall in pH (Davis et al., 1983; Figure 7A). Studies have demonstrated that VE is exaggerated in patients with mtDNA mutation during exercise (Haller and Lewis, 1984; Haller et al., 1989; Flaherty et al., 2001; Taivassalo et al., 2003; Heinicke et al., 2011), and the level of VE correlated with the muscle mtDNA mutation load (Taivassalo et al., 2003; Taivassalo and Haller, 2005). Increased motor unit recruitment and excessive build-up of ADP, lactate, and other metabolites in exercising muscle may very likely be responsible for the excessive VE found in patients with mtDNA mutation (Figure 7B). The few studies that have reported VE adaptations to aerobic exercise in patients with mtDNA mutation, overall, did not find a change in VE with aerobic training, which indicates that factors responsible for enhanced VE in patients with mtDNA mutation do not change with aerobic training. Thus, the excessive VE in patients with mtDNA mutations during exercise may be one of the factors responsible for premature fatigue. Only one study has reported VE during constant workload test, and found that VE rate decreased after 12 weeks of training, indicating that aerobic training might have a positive effect on the otherwise excessive VE rate seen in patients with mtDNA mutation (Cejudo et al., 2005).

## The Impact of Aerobic Training: Fuel Metabolism During Exercise in Patients With mtDNA Mutation

During exercise the relative contribution of carbohydrate and fat for energy (ATP) synthesis varies with intensity and duration of the exercise bout (Saltin and Astrand, 1993; Hawley et al., 2011). From rest to exercise intensity of 50% of VO_2__max_, the principle substrate for ATP production is free fatty acids (FFA) (Saltin and Astrand, 1993). From exercise intensity of 50% of VO_2__max_, oxidation of carbohydrate increases linearly with increase in exercise intensity, but from exercise intensities beyond 85% of VO_2__max_, anaerobic metabolism becomes increasingly important until peak VO_2_ (Van Hall et al., 2003). Since oxidation of glucose releases more ATP per molecule of O_2_ that is utilized compared to oxidation of FFA, it has been speculated if carbohydrate could be the preferred energy substrate in patients with mtDNA mutation in skeletal muscle. However, two studies have shown that FFA and carbohydrate are oxidized to the same extent at rest and during exercise as that seen in healthy individuals (Jeppesen et al., 2009b, 2013). In healthy individuals, aerobic training changes fuel turnover from carbohydrate toward increased fatty acid oxidation (Bylund et al., 1977; Saltin and Astrand, 1993). This change is met by an increase in mitochondrial biogenesis, including increased respiratory chain enzyme levels (Holloszy, 1975; Andersen and Saltin, 1985; Hoppeler and Fluck, 2003), which lowers muscle lactate production and respiratory exchange ratio (Davis et al., 1983). The rate and slope of these changes depend on pre-training status, duration, and intensity of training. No study has, to date, investigated changes in carbohydrate and FFA turnover in patients with mtDNA mutation. However, since FFA and carbohydrate turnover is the same at rest and during exercise in patients with mtDNA mutation compared to healthy subjects, training-induced changes in fuel turnover would not be expected to be different than that found in healthy subjects.

**FIGURE 8:**
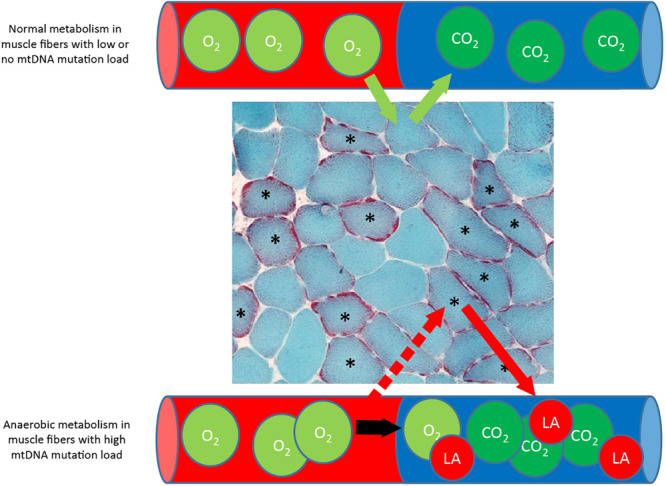
Metabolism in muscle fibers with low and high mtDNA mutation load. Muscle section from patients with mtDNA mutation, stained with Gomori Trichrome staining showing multiple ragged red (^∗^) muscle fibers that indicate accumulation of dysfunctional mitochondria due to high mtDNA mutation load. During exercise, fibers with a high mtDNA mutation load is dependent on anaerobic metabolism alone due to mitochondrial dysfunction, while adjacent muscle fibers with low or no mtDNA mutation load will be able to oxidize fat and glucose and take up lactate (La) for oxidation.

In patients with mtDNA mutation, resting plasma lactate levels correlate with mtDNA mutation load in skeletal muscle (Jeppesen et al., 2003), which has prompted the idea that net lactate release from skeletal muscle depends on oxidative capacity in skeletal muscle. However, only one study has investigated lactate turnover in patients with mtDNA mutation. This study demonstrated that the capacity to oxidize lactate is not limited in patients with mtDNA mutations, not even in patients with high level of mtDNA mutation in skeletal muscle (Jeppesen et al., 2013). The authors argued that the substantial high lactate level found at rest and during exercise in patients with mtDNA mutation may be a result of a constant production of lactate in fibers with high levels of mtDNA mutation along with intact lactate oxidation capacity in muscle fibers with no or low levels of mtDNA mutation (Figure 8). This hypothesis is related to the heterogeneous distribution of mtDNA mutation load among skeletal muscle in patients with mtDNA mutation irrespective of type I or type II fibers resulting in a situation where some fibers may rely solely on anaerobic glycolysis, while adjacent muscle fibers with lower mtDNA mutation load will be able to oxidize lactate (Figure 8). Siciliano et al. (2000, 2012) investigated the effect on aerobic training on lactate levels during constant workload test, and in one study, the concomitant catecholamine production was investigated. The authors showed that patients with mtDNA mutation had a lower plasma lactate level during a constant workload test after 10 weeks of aerobic training compared to healthy subjects. Interestingly, the authors found that the catecholamine levels decreased to the same extent as those seen in healthy subjects, indicating that the higher catecholamine level was not the driving factor for exaggerated lactate level found in patients with mtDNA mutation (Siciliano et al., 2012). Instead, the study implied that decreased lactate level after aerobic training in patients with mtDNA mutation could be a result of increased capacity to oxidize lactate on skeletal muscle level.

The premature fatigue that is often seen in patients with mtDNA mutation has been linked to the excessive build-up of lactate (Dengler et al., 1996; Finsterer et al., 1998). However, in one study where resting lactate level was reduced pharmacologically, exercise capacity did not improve in patients with mtDNA mutation. This finding indicated that premature fatigue in patients with mtDNA mutation may not be related to plasma lactate levels (Vissing et al., 1998). Another potential explanation for premature fatigue could be that a relative higher fraction of muscle fibers is recruited during exercise in patients with mtDNA performing the same workload as healthy subjects. This hypothesis is based on the heterogeneous distribution of mtDNA mutation load among fibers in the same muscle (Figure 8). Thus, muscle fibers with a high mtDNA mutation load rely heavily on anaerobic glycolysis and are, thus, prone for depleting glycogen storage fast, which inevitably result in recruitment of additional muscle fibers for muscle contraction (Figure 8). The higher skeletal muscle fiber recruitment result in a sense of fatigue and, thus, could induce premature fatigue in patients with mtDNA mutation compared to healthy subjects exercising at the same relative workload (Saltin, 1981). Training studies investigating the effect of aerobic training on resting and peak-exercise-induced lactate levels have shown that exercise-induced fatigue was reduced after 8–14 weeks of aerobic training (Taivassalo et al., 1998, 2006; Siciliano et al., 2000; Cejudo et al., 2005; Porcelli et al., 2016). At the same time, peak-lactate level remained unchanged (Siciliano et al., 2000, 2012; Cejudo et al., 2005; Jeppesen et al., 2006b, 2009a; Porcelli et al., 2016) or was higher (Taivassalo et al., 1998, 2001) in patients with mtDNA mutation. This finding underscores that the absolute plasma lactate level does not, itself, induce premature fatigue in patients with mtDNA mutations.

## Impact of Aerobic Training: Adaptation of Mitochondrial Volume and Mitochondrial DNA

Although only a few studies have examined the effect of aerobic training on mitochondrial content directly (Hoppeler and Weibel, 1998; Hoppeler and Fluck, 2003), it is widely accepted that mitochondrial volume density increases with aerobic training. Mitochondrial content is usually assessed with indirect measures like citrate synthase, cardiolipin, porin, and mtDNA copy number. Citrate synthase, which is the rate-limiting step in the Krebs cycle, correlates closely with oxidative capacity (Schwerzmann et al., 1989; Kiens et al., 1993; Rasmussen et al., 2001; Jacobs et al., 2013); cardiolipin, which is a phospholipid located in the inner mitochondrial membrane, has been found to be a good biomarker of the total mitochondrial cristae surface area in human skeletal muscle (Pfeiffer et al., 2003; Larsen et al., 2012); and porin, which is the most abundant protein of the mitochondrial outer membrane, correlates with the mitochondrial volume in skeletal muscle (van Moorsel et al., 2016). It has been a general notion that mtDNA copy number is replicated along with an increase in mitochondrial content, and therefore, mtDNA content has also been used as a marker of mitochondrial volume (Menshikova et al., 2006; Civitarese et al., 2007; Balakrishnan et al., 2010; Perry et al., 2010; Zoladz et al., 2013). It has been a concern if shorter mtDNA copies (mutated mtDNA) could have a replicative advantage over larger mtDNA copies (wild type) in patients with mtDNA mutations (Moraes et al., 1989; Larsson et al., 1990; Fu et al., 1996; Weber et al., 1997; Chinnery and Samuels, 1999), which inevitably would result in increase in mtDNA mutation load in skeletal muscle with continuous training. When a study demonstrated that mtDNA mutation load increased in six of nine patients after 14 weeks of cycle exercise, the safety of aerobic exercise in patients with mtDNA mutation was questioned (Taivassalo et al., 2001). Only one study has investigated mtDNA copy number in trained vs. untrained limb of the same subjects and demonstrated that mtDNA copy number does not change with aerobic training (Fritzen et al., 2019). This finding demonstrates that changes in mitochondrial volume do not result in changes in mtDNA copy number and, thus, prove that mtDNA mutation load will not increase in skeletal muscle in patients with mtDNA mutations because of increase in oxidative capacity. In line with this, none of the following training studies conducted in patients with mtDNA mutations found any evidence of change in mtDNA mutation load, indicating that aerobic training in patients with mtDNA mutations is safe (Jeppesen et al., 2006b, 2009a; Taivassalo et al., 2006).

**FIGURE 9:**
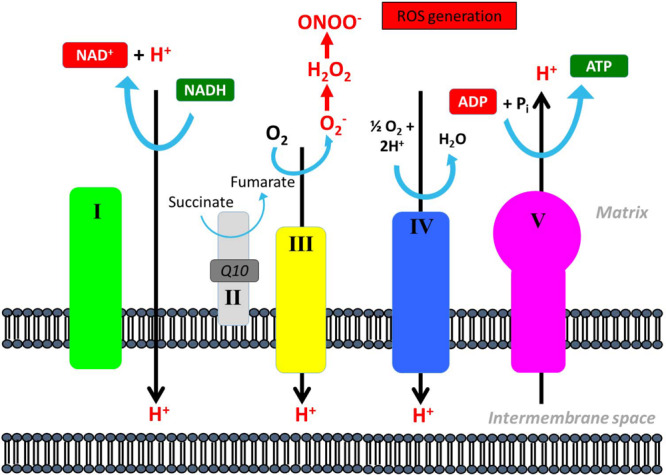
Reactive oxidative species production in the mitochondrial respiratory chain. Reactive oxidative species (ROS) can be generated through conversion of oxygen (O_2_) to superoxide anion (O2^–^) that can be converted further into peroxynitrite (ONOO^–^) that is a highly reactive species and can oxidize DNA, proteins, and lipids and interfere with important vascular signaling pathways.

It is a general concern that patients with mtDNA mutation, due to uncoupling of complexes in the respiratory chain, produces higher levels of reactive oxidative species (ROS) than healthy patients with intact mitochondria (Figure 9). ROS is harmful to the cell due to induction of mtDNA mutation, activation of mitochondrial permeability pore and direct damage to lipid membranes (Lu et al., 2003; Ikawa et al., 2009; Liu et al., 2009; Wu et al., 2010; Siciliano et al., 2012). During exercise, there is an increased flux through the respiratory chain, and therefore, studies have questioned the safety of long-term training in patients with mtDNA mutations (Adhihetty et al., 2007; Siciliano et al., 2012). Only two studies have directly investigated differences in oxidative stress and compensatory mechanisms related to training in patients with mtDNA mutation compared to healthy subjects (Adhihetty et al., 2007; Siciliano et al., 2012). One study found that there was an increased ROS production in patients with mtDNA mutation, but this was counteracted by compensatory mechanisms. These compensatory mechanisms included increased antioxidant levels, increased DNA repair capacity, and elevated levels of Bcl-2, which serve as a protective mechanism by lowering pro-apoptotic proteins (Adhihetty et al., 2007). However, the authors found that there was a reduced expression of DNA repair machinery along with an increased oxidative damage in skeletal muscle after 14 weeks of aerobic training (Adhihetty et al., 2007). Siciliano and authors showed that 10 weeks of aerobic training resulted in a decrease in blood lipoperoxide, which indicated that training induced a “one-step scaled down” on the conventional four-step scale adopted for oxidative stress (Siciliano et al., 2012). Taken together, training seems to induce factors that counteract potentially harmful effects of training on mitochondria in patients with mtDNA mutation, but whether the harmful effect is counterbalanced on a long term still remains unclear and warrants further investigation.

## Impact of Aerobic Training: Quality of Life and Functional Testing

A typical consequence of low oxidative capacity in patients with mtDNA mutation is impaired physical performance including difficulties in walking. Thus, it seems straightforward that along with indices of training-induced increases in oxidative capacity, quality of life, and functional assessment should also be performed. However, only a few studies have investigated the impact of training on quality of life (Taivassalo et al., 1998, 2001, 2006; Cejudo et al., 2005; Porcelli et al., 2016). Moreover, only one study aimed to convert an increase in oxidative capacity into clinical meaningfulness by a functional test (Cejudo et al., 2005). Improvements in quality of life ranged from no-change to 25% improvement after 8–14 weeks of aerobic training. Interestingly, an improvement of 25% in oxidative capacity did not result in a higher physical activity level during or after the training period (Porcelli et al., 2016), indicating that increase in oxidative capacity of 25% not necessarily translates into a more physical active lifestyle.

## Perspective

All individuals, even sedentary, experience repeated periods where physical activity level differs. In line with this, patients with mtDNA mutation also experience periods with a higher physical activity than others. With the potential risk that physical exercise may increase mtDNA mutation load, physical exertion could potentially result in mitochondrial dysfunction with time in patients with mtDNA mutation. No study has, to date, investigated if the level of new mtDNA mutations increases with time in patients with mtDNA mutation and whether this could be related to the level of physical activity. A cross-sectional study of a large cohort of patients with the common 3,243 A > G mutation have indicated that the inherited mtDNA mutation does no increase with time (Frederiksen et al., 2006). However, this finding does not rule out that patients with inherited mtDNA mutations may be more prone to develop new mtDNA mutations with time. In theory, an increase in new mtDNA mutations and, thus, increased dysfunctional mitochondria with time, could be the driving factor for the progressive nature of disease in patients with mtDNA mutations. Thus, one scope for future long-term training studies could be assessment of new mtDNA mutations along with assessment of the level of ROS, mtDNA repair activity, and antioxidant levels.

## Author Contributions

TJ drafted the manuscript.

## Conflict of Interest

The author declares that the research was conducted in the absence of any commercial or financial relationships that could be construed as a potential conflict of interest.
